# Spinal Anaesthesia for Laparoscopic Cholecystectomy in Parkinson's Disease

**Published:** 2018-06-30

**Authors:** Binod Gautam, Bikash Baral

**Affiliations:** 1 Department of Anaesthesia and Intensive Care, Kathmandu Medical College, Sinamangal, Kathmandu, Nepal

**Keywords:** *Laparoscopic cholecystectomy*, *Parkinson's disease*, *spinal anaesthesia*

## Abstract

Parkinson's disease, a neurodegenerative disorder, presents with resting tremor, muscle rigidity and bradykinesia. Affecting multiple organ-systems, it's an important cause of peri-operative morbidity. General anaesthesia may deteriorate cardio-pulmonary and neuro-cognitive functions; moreover, medications used may interact with anti-parkinsonian agents. Spinal anaesthesia is usually avoided in Parkinson's disease. However, it offers neurologic monitoring and less surgical stress response and avoids complications of general anaesthesia. This case report aims to demonstrate application of spinal anaesthesia for laparoscopic cholecystectomy in a Parkinson's elderly with pulmonary dysfunction and anticipated difficult airway management. Sensory blockade of third thoracic dermatome was achieved. Bupivacaine was instilled intra-peritoneally. Surgery was smooth at low intra-abdominal pressure. Regular Paracetamol provided satisfactory post-operative analgesia. Single episode of post-operative vomiting was effectively managed. Without deterioration, patient was discharged from hospital on third day. Spinal anaesthesia is a valid technique for laparoscopic cholecystectomy in needy patients with multiple peri-operative risks.

## INTRODUCTION

Parkinson's disease (PD), a neurodegenerative condition, presents with movement disorder, muscle rigidity, dementia and depression. Varied systemic associations and drug interactions during general anaesthesia (GA) make Parkinson's patients prone to cardio-pulmonary and neuro-cognitive complications.^1,2^

Spinal anaesthesia (SA) is usually avoided in PD with the fear of clinical deterioration. However, it avoids complications and potential drug interactions associated with GA; and offers easy neurologic monitoring and early recovery. SA has been proved advantageous in healthy and high-risk patients undergoing laparoscopic cholecystectomy (LC).^3–5^ We report a Parkinson's elderly with difficult airway and pulmonary dysfunctionwho underwent LC uneventfully under SA.

## CASE REPORT

A 65 years female weighing 72 kg was planned for LC for calculus cholecystitis. She had Parkinson's disease and was taking oral medications (Levodopa 100 mg plus Carbidopa 25 mg thrice a day) since one and half years. She was also taking Trifluoperazine 2.5 mg, Trihexyphenidyl 1 mg and Escitalopram 10 mg once a day orally for severe depressive episode with psychotic symptoms since three months. On examination she had tremors, bradykinesia, stooped posture and rigidity. She could comprehend well but she had a decreased coughing ability. There was no sialorrhea, orthostatic hypotension or bladder dysfunction.

Her airway examination showed Mallampatti score of three and severely restricted neck extension leading to an anticipation of difficult airway management. Chest auscultation revealed decreased air entry on the basal lung regions bilaterally. Other systemic examinations were within normal limits. Her liver function tests were slightly deranged but other routine investigations were normal. She was unable to perform pulmonary function test on a spirometer. The patient was pre-medicated with Ranitidine and Domperidone orally on the night before and at morning of surgery. She was fasted for eight hours for solid food and was allowed to drink clear liquid till two hours before surgery. Patient received her usual morning dose of Levodopa and Carbidopa in the pre-operative room.

Standard monitors were utilized in the operating room. Her baseline vitals were heart rate (HR) at 98/minute, blood pressure (BP) of 163/86 mmHg and Oxygen saturation of 94% in room air. Intravenous (IV) access was achieved with a 16 Gauge cannula on right hand. She received 500 ml Ringer's Lactate, Paracetamol 1 gram, Diclofenac 75 mg, Ceftriaxone 1 gram and Midazolam one mg IV. Oxygen 6 litres/minute was provided via Hudson's mask. Provisions for emergency airway management, fiberoptic bronchoscope system and percutaneous tracheostomy set and personnel for tracheostomy were standby.

She was placed in left lateral decubitus position. Three ml 2% Lignocaine was used for skin infiltration at L_2_/L_3_ intervertebral space. 25 Gauge Quincke spinal needle was inserted into subarachnoid space. With confirmed free flow of cerebrospinal fluid, 3 ml 0.5% hyperbaric Bupivacaine was injected over 30 seconds. The procedure went uneventful and patient was made to assume horizontal resting position with table tilt at 10 degrees head down.

At two minutes of completion of subarachnoid injection, her BP measured at 77/47 mmHg with HR of 68. Mephentermine 9 mg was given IV, head end was slightly elevated and a bolus of 300 ml Ringer's Lactate was infused rapidly. BP after a minute measured at 144/87 with HR of 92. Sensory blockade level was found to be at the third thoracic dermatome.

Surgery was performed using three-trocar and open-laparoscopy technique with intra-abdominal pressure limit set at 10 mmHg and flow rate of carbon dioxide oftwoto threelitres/minute. After creation of surgical ports, 30 ml 0.25% Bupivacaine was sprayed on to the undersurface of right hemi-diaphragm and over the gall bladder plus Callot's triangle. Patient was repeatedly asked if she was comfortable and she replied positively. Nasogastric tube insertion was not required. Patient's monitored parameters remained stable throughout the surgery.

She received a total of 1700 ml crystalloids for 35 minutes’ duration of surgery. At the end, sensory blockade was still at third thoracic dermatome and monitor showed her HR at 82/min, BP of 163/90, respiratory rate of 20/min and Oxygen saturation of 100% before her transfer to the post-anaesthesia care unit.

Patient had an episode of vomiting five minutes after completion of surgery with the normal hemodynamic parameters. She was given IV Ondansetron 8 mg in 100 ml normal saline over 10 minutes. Dexamethasone 8 mg was efficient to wane her symptoms of nausea. Patient was kept on supplemental oxygen with head end elevated at 15 degrees. Regular IV Paracetamol 1 gram three times a day was sufficient for pain relief and she did not miss a single dose of her pre-operative medications. Chest physiotherapy could be started 10 hours after surgery and post-operative course being uneventful, shewasdischarged from hospital on 3^rd^ day. Neurological and psychiatric consultation instructed her tocontinuepre-operative drug regime.

## DISCUSSION

The problems associated with this elderly patient which included Parkinson's disease, difficult airway and pulmonary dysfunction with atelectasis made her to be categorized as American Society of Anesthesiologists’ physical status class three. Considering the pulmonary complications and potentially serious drug interactions associated with GA, and our experience in SA to conduct LC, we felt obliged to do the same.

Parkinson's disease, relatively a common neurodegenerative disorder, classically presents with resting tremors, muscle rigidity and bradykinesia. With the progressive and selected loss of dopaminergic neurones of the substantianigra and the resultant imbalance of neurotransmitters favoring gammaaminobutyric acid over activity is responsible for the clinical picture.^1^ The medical basis of treatment is to restore the balance by either increasing Dopamine or dopamine-like activity or reducing cholinergic activity within the brain. Particular anaesthetic implications in PD include old age with co-existing diseases, various drug effects and interactions, airway abnormalities, and many alterations in the pulmonary, cardiovascular, autonomic and neurologic systems.^1–2^ The ongoing therapy should always be continued during the perioperative period, as in our patient, to avoid abrupt withdrawl with the deterioration of symptoms.^2^

SA is generally not chosen for Parkinson's patients, the basic concern being the possibility of exacerbation and clinical deterioration. There is paucity of reports with the use of SA in Parkinson's patients but there is no definitive recommendation whether SA poses a contraindication.^6^ On the other hand, the need for multiple medications with GA and PD-related complications make SA more advantageous. SA also allows early identification of exacerbation, better pain management, lower incidence of nausea and vomiting and attenuation of surgical stress response which ultimately allow early post-operative recovery.^1^ However, tremors and severe dyskinesia, at times, pose difficulties including mechanical performance of SA, interference with monitoring devicesand surgical procedure itself, and might make GA preferable. But intravenous induction agents, inhalational agents, neuromuscular blocking agents and Opioids used for GA could precipitate the symptoms as well as delay the diagnosis of exacerbation. Besides the pulmonary complications, we aimed to avoid drug interactions and unwanted effects of conventional endotracheal GA in our patient.

Respiratory function in our patient was compromised by bradykinesia and muscle rigidity as well as by sputum retention. Her advanced age, impaired coughing ability and atelectasis were definitive predictive factors for pulmonary complications. Peri-operative aspiration pneumonia, post-extubation laryngospasm, chest infectionand respiratory failure have been reported in Parkinson's patients after anaesthesia.^7–9^ In this scenario, we preferred SA which avoided airway instrumentation or respiratory support and also allowed early mobilization and chest physiotherapy that could minimize pulmonary complications.

The issue of anaesthetic management of a patient with difficult airway guides us to one of two approaches. The choice between securing a potential difficult airway versus evading it by employing regional anaesthesia is influenced mostly by patients’ co-morbidities, and familiarity of anaesthesiologist with the particular anaesthetic technique. These days awake fiberoptic intubation is considered to be the safest option and is being employed ever more to tackle anticipated airway difficulties. However, this approach is sensible only if GA with muscle relaxant is an absolute necessity for the surgical procedure in question and as properly applied SA offers several advantages compared to GA for conducting LC, we opted for the same.^4^

Autonomic dysfunction in Parkinson's patients can produce diverse manifestations including orthostatic hypotension, sialorrhea, constipation, incontinence and excessive sweating.^1,2^ It can lead to sudden or exaggerated response to central neuraxial blockade. The rapid ascent of spinal blockade and hypotension witnessed in our patient could have been attributed by undiagnosed autonomic dysfunction, in addition to her advanced age, relative hypovolemiaand medications. Parkinson's patients are predisposed to dysphagia, gastric stasis and medications-induced nausea and vomiting.^2^ In addition, laparoscopic surgery did attribute to her vomiting, which was however easily managed with Ondansetron and Dexamethasone. Despite having neuropsychiatric symptoms of depression and psychotic episodes she did not develop post-operative confusion and delirium, the incidence of which is quite high after GA in these patients.^1–2^

We will never know what would have been the course and outcome of our patient had she received GA; however, with our approach she had no discomfort and was communicating well during surgery, oriented at the end and was comfortable throughout her hospital stay. She didn't have her clinical condition deteriorated and could be discharged early from the hospital. Gentle surgical technique and minimal stress anaesthetic technique offered the easily manageable post-operative pain with no requirement for Opioids. There is no “cook-book” anaesthetic regime for such patients and because of personal familiarity and experience we relied on SA which provided adequate surgical anaesthesia and a good quality post-operative analgesia and avoided the potentially serious complications of GA.

Spinal anaesthesia with multimodal analgesic techniques is a suitable option to provide an effective condition for elective laparoscopic cholecystectomy in Parkinson's patients without exacerbating the disease symptoms. It avoids the peri-operative risks associated with the pre-existent abnormal pulmonary function and anticipated airway might make general anaesthesiaa high-risk procedure.

**Consent:** JNMA Case Report Consent Form was signed by the patient and original is attached with the patient chart.
